# Performance Evaluation of Construction Companies Using Integrated Entropy–Fuzzy VIKOR Model

**DOI:** 10.3390/e23030320

**Published:** 2021-03-08

**Authors:** Weng Siew Lam, Weng Hoe Lam, Saiful Hafizah Jaaman, Kah Fai Liew

**Affiliations:** 1 Department of Physical and Mathematical Science, Faculty of Science, Kampar Campus, Universiti Tunku Abdul Rahman, Jalan Universiti, Bandar Barat, Kampar 31900, Malaysia; whlam@utar.edu.my (W.H.L.); liewkf@utar.edu.my (K.F.L.); 2 Department of Mathematical Sciences, Faculty of Science and Technology, Universiti Kebangsaan Malaysia, UKM, Bangi 43600, Malaysia

**Keywords:** entropy, fuzzy VIKOR, multi-criteria decision making, financial ratio, research framework

## Abstract

The construction sector plays an important role in a country’s economic development. The financial performance of a company is a good indicator of its financial health and status. In Malaysia, the government encourages the construction industry to develop an advanced infrastructure related to health, transport, education and housing. In view of the COVID-19 pandemic, the operations and financial performance of construction sector companies have been affected recently. Additionally, uncertainty plays a vital role in the multi-criteria decision-making (MCDM) process. Based on previous studies, there has been no comprehensive study conducted on the evaluation of the financial performance of construction companies by integrating entropy and fuzzy VIKOR models. Therefore, this paper aims to propose an MCDM model to evaluate and compare the financial performance of construction companies with an integrated entropy–fuzzy VIKOR model. A case study is carried out by evaluating the listed construction companies in Malaysia with the proposed model. The findings of this paper indicate that the company ECONBHD achieves the best financial performance over the study period. The significance of this paper is to determine the priority of the financial ratios and ranking of the construction companies with the proposed entropy–fuzzy VIKOR model.

## 1. Introduction

COVID-19, which was declared a pandemic by the World Health Organization (WHO) in March 2020 and is still tempestuous, has caused dramatic losses globally, both among humans and economically. Its unknown magnitude, impact and duration pose a vicious cycle of ousting businesses, jeopardizing consumer confidence and tightening financial conditions leading to losses in jobs and investment. Globally, nations reacted to the pandemic by taking precautionary measures, such as banning travelling and imposing certain kinds of movement control order (MCO), shutting down government and private premises and imposing total or partial lockdowns. Pre-pandemic, these revolutionary actions would be considered unthinkable, but it is now termed the “new normal”, where workers are forced to work from home, food is delivered and meetings and business dealings are conducted online.

The movement control order and social distancing observed have severely damaged the construction industry. For emerging nations, such as Malaysia, the construction sector plays a vital role in realizing their goals to become modernized and have a strong industrialized economy. The construction sector provides great support to the aggregate economy by forward and backward linkages with other sectors of the economy. It also contributes to generating huge employment in the economy [1]. In Malaysia, the construction industry contributed 5.9% to the gross domestic product (GDP) in 2017, whereas total industry growth for the year stood at 6.7%. The government’s vision 2020 project will also boost subsector construction projects in the coming years to improve the country’s tourism infrastructure and transport network and also increase the volume of renewable projects. In addition, the government strives to address the country’s housing shortage because it can assist the industry in expanding over the next five years [2]. During the period of 2016 to 2018, the construction industry suffered a decline of 60%. Moreover, in 2016, the industry’s annual turnover reached a peak of RM 273 billion, and this figure declined to RM 106 billion in 2018 [3]. According to a statistic reported, the movement control order’s impact would be immense to contractors [3]. Due to the MCO, projects are being halted, suspended, or even cancelled. Furthermore, the movement of workers at construction sites has been cited to be the main cause of COVID-19 transmission at some workplace clusters. The pandemic has also shown that the construction sector depends heavily on unskilled foreign labor due to low technology adoption.

The COVID-19 pandemic has immensely affected the financial performance of sectors such as tourism, travel, aviation, retail and construction [4]. It is crucial for companies and stakeholders to understand the financial impacts caused by the pandemic. For existing and potential investors, relevant decisions are made based on the understanding of published financial reports and industry hearsay. The financial data reported under GAAP or IFRS, such as operating income, reflect an accurate view of past performance. The industry unpublished hearsay provides the required additional information to understand the performance of key drivers as a basis to predict future performance. Studies at the microlevel are equally important to studies conducted at the macrolevel. Focusing on firm level financial data allows us to quantify the impact of the pandemic on the performance of individual companies and certain industries. Shen et al. [5] found that the COVID-19 outbreak has had a significant negative impact on the performance of Chinese firms, reducing total revenue and investment.

Financial performance evaluation is important for companies in a challenging and competitive environment, especially during the current COVID-19 pandemic. Construction companies should understand their current financial performance, because it is important to all companies in the same field. Financial ratio is a good indicator of the financial performance of companies, as it can disclose their financial strengths and weaknesses [6]. Financial ratios of return on equity (ROE), return on asset (ROA), earnings per share (EPS), debt to equity ratio (DER), debt to assets ratio (DAR) and current ratio (CR) are the important financial ratios used to measure the financial performance of companies [6,7,8,9]. The ranking of companies is significant and important in the financial performance evaluation because companies wish to know their ranking among their competitors in the same field or sector for benchmarking purposes [6]. Companies can implement the relevant strategies to improve their financial performance based on their current financial status and ranking.

Multi-criteria decision making (MCDM) is an important process of decision analysis to select the best alternative by considering multiple decision criteria or factors [10]. Decision criteria and decision alternatives are two important elements in determining the performance and ranking of alternatives. Evaluation of companies’ financial performance is an MCDM problem, since the decision-making process involves multiple financial ratios. Ginevicius and Podvezko [11] studied the financial performance of construction companies with the proposed VIseKriterijumska Optimizacija I Kompromisno Resenje (VIKOR) model. In their study, the financial performance of companies was evaluated based on the financial ratios. According to the result, the financial performance and ranking of construction companies can be determined based on the optimal solution of the VIKOR model. Abdel-Basset et al. [6] measured the financial performance of manufacturing companies in Egypt with the VIKOR model by focusing on multiple financial ratios, such as ROE, ROA, DER, DAR and CR.

In today’s challenging world, uncertainty plays a vital role in decision-making processes. Therefore, it is essential to handle uncertain information in data. The entropy weight method was introduced by Shannon, which assigns the objective weight for each decision criterion based on data analysis [12]. Due to the uncertainty of signals in information sources, Shannon considered the entropy into information theory [13]. The entropy weight method is an efficient tool to measure uncertain information [14]. Due to the importance of the entropy weight method in the decision-making process, the researchers considered the entropy weight method in the evaluation [15,16,17]. The entropy weight method was mainly used to calculate the relative weight between the decision criteria [18]. The entropy weight of the decision criteria can be determined based on the data obtained. The entropy weight method is important in obtaining the information entropy of the decision criteria and the degree of difference of the decision criteria. This information is required to measure the weight of the decision criteria and effective information contained in the known data. The entropy weight method has been applied in various fields, such as supplier selection [19], prediction of the unfrozen water content [20], building material supplier selection [18], jump volatility spillover network [21], children’s physical activity and human development index [22], fog-haze factor assessment [23] as well as test case prioritization [24].

The VIKOR model is adopted to measure the compromise solution that is the farthest from the negative ideal solution (NIS) and closest to the positive ideal solution (PIS) and rank the decision alternatives. Due to the ambiguity and uncertainty of the financial data [25], the fuzzy VIKOR model is proposed in this study to tackle the MCDM problem with non-commensurable and contradictory criteria in a fuzzy environment [26,27,28]. The fuzzy VIKOR model’s central idea is based on the ideal solution of merit function *Q*. The fuzzy VIKOR is a powerful and useful model in solving multi-criteria group decision-making problems. The fuzzy VIKOR model has outstanding advantages in evaluating the alternatives over conflicting criteria. Carlsson and Fuller [29] presented the fuzzy method in the evaluation and decision-making process. Since decision makers’ judgments are usually imprecise when choosing an alternative with respect to multiple decision criteria, the fuzzy concept is incorporated into the MCDM process [30,31]. The fuzzy method is robust because it helps to express the fuzziness, uncertainties, vagueness and imprecision during the decision-making process [32]. Hence, it is important that the fuzzy method is integrated with the entropy–VIKOR model in order to obtain more concrete and realistic results [33]. The fuzzy VIKOR model has been widely used in the field of development of indices and wells ranking systems [34], sustainable supplier selection [35], prioritization of watershed reforestation [36], energy systems assessment [37], water consumption pattern [38], construction project management [39], residential demand response [40] and water resource planning [41].

Many industries, including construction companies, have been greatly affected by the COVID-19 pandemic. According to previous studies, there is no research that has been carried out on the investigation of financial performance among construction companies by integrating entropy and fuzzy approaches into the VIKOR model. Previous studies have proposed the VIKOR model to assess the financial performance of construction companies without considering the entropy weight and fuzzy methods [6,11]. The VIKOR model has limitations in setting decision criteria weight, since its weight is subjectively judged by the decision maker. Hence, the literature left a research gap to incorporate the entropy weight and fuzzy methods for the evaluation of the financial performance of construction companies. The primary goal of this paper is to propose an MCDM model, namely, an entropy–fuzzy VIKOR model, to assess and compare the financial performance of construction companies. The proposed model is illustrated with a real case study in Malaysia by evaluating the financial performance of construction companies. Additionally, the comparison of empirical results is performed between the VIKOR model and the proposed model. The VIKOR model has been presented in previous studies for financial performance evaluation. Therefore, the reason for the comparison of empirical results is to study the impact of the integration of entropy and fuzzy approaches in the VIKOR model. The remainder of this manuscript is structured as follows. Section 2 presents the materials and methods, which include the research development and methodology of the proposed entropy–fuzzy VIKOR model as well as the VIKOR model. Section 3 demonstrates the empirical results of the proposed entropy–fuzzy VIKOR model. Next, we present the comparison of empirical results between the VIKOR model and the proposed model. Lastly, concluding remarks based on the research findings are enumerated in the last section of this article.

## 2. Materials and Methods

### 2.1. Research Development

In this research, we propose an MCDM model, namely, the entropy–fuzzy VIKOR model, to evaluate and compare the financial performance of construction companies. The proposed model consists of two stages as follows:

Stage 1: Determine the weights of decision criteria (financial ratios) with the entropy weighting method;

Stage 2: Compare and rank the decision alternatives (construction companies) with the fuzzy VIKOR model.

Table 1 depicts the proposed research framework to evaluate the financial performance of construction companies with the integrated entropy–fuzzy VIKOR model.

Table 1 depicts the proposed research framework, which comprises the main objective, decision criteria and decision alternatives for the assessment of construction companies in terms of financial performance. In this paper, important financial ratios, such as CR, DAR, DER, EPS, ROA and ROE, were considered as the decision criteria [42,43,44,45,46]. The proposed model is illustrated with a real case study by evaluating the listed construction companies in Malaysia [47] for the period of 2015 to 2019 with the proposed model. In addition, the comparison of empirical results was performed between the proposed model and VIKOR model. The VIKOR model has been employed by previous researchers to evaluate the financial performance of construction companies [11]. The methodology of the proposed model and VIKOR model is presented in the following Section 2.2 and Section 2.3, respectively.

### 2.2. Proposed Entropy–Fuzzy VIKOR Model

In the first stage, the entropy weight method was employed to identify the objective weights of the financial ratios because it can avoid the subjectivity of weight selection [48,49,50]. Moreover, the entropy weight method is able to fully utilize the sample data to obtain the importance weight of the financial ratios [37].

In the second stage, the fuzzy VIKOR model was proposed to evaluate, compare and rank the decision alternatives (construction companies). The Fuzzy VIKOR model is a decision tool that deals with decision problems with non-commensurable and contradictory attributes by considering fuzziness and uncertainty. Moreover, the fuzzy VIKOR model was adopted to obtain the compromise solution that is the farthest from the NIS and closest to the PIS and determine the ranking of the decision alternatives by using the individual maximum regret value and group utility value. The merits of the fuzzy VIKOR model are to rank and select the alternatives with conflicting and inconsistent criteria [51,52,53,54]. Assuming that each decision alternative is assessed based on multi-criteria functions, the compromise ranking was identified by comparing the measure of closeness to the ideal [26,27,55]. The fuzzy set theory was firstly proposed by Zadeh to describe fuzziness and uncertainty [56]. The entropy–fuzzy VIKOR model is presented as follows:

Step 1: Computation of financial ratio weight via the entropy weight method. Calculate the proportion “*p_ij_*” of the index value of alternative *m* under criterion *n*.

(1) $$p_{ij}=\frac{x_{ij}}{\sum_{i=1}^{m}x_{ij}},i=1,2,\ldots ,m,j=1,2,\ldots ,n$$

where $x_{ij}$ represents the *j*th criterion value of the *i*th alternative of the initial matrix *D*.

Step 2: Determination of the entropy “*e_j_*” of criterion *n*.

(2) $$e_{j}=-k\sum_{i=1}^{m}p_{ij}.\ln(p_{ij}),j=1,2,\ldots ,n$$

where $k=\frac{1}{\ln(m)}$.

Step 3: Calculation of the entropy weight “*w_j_*” of criterion *n*.

(3) $$w_{j}=\frac{1-e_{j}}{\sum_{j=1}^{n}\left(1-e_{j}\right)},j=1,2,\ldots ,n$$

Step 4: Establish the fuzzy decision matrix on the basis of triangular fuzzy numbers for the decision alternatives with respect to the criteria [57,58,59].

(4) $$D=\left[\begin{array}{ccccc} {\tilde{x}}_{11} & \cdots & {\tilde{x}}_{1j} & \cdots & {\tilde{x}}_{1n}\\ \vdots & & \vdots & & \vdots \\ {\tilde{x}}_{i1} & \cdots & {\tilde{x}}_{ij} & \cdots & {\tilde{x}}_{in}\\ \vdots & & \vdots & & \vdots \\ {\tilde{x}}_{m1} & \cdots & {\tilde{x}}_{mj} & \cdots & {\tilde{x}}_{mn} \end{array}\right]$$

where ${\tilde{x}}_{ij}=(a_{ij},b_{ij},c_{ij})$, i=1,…,m,j=1,…,n. *m* refers to the number of decision alternatives. *n* refers to the number of decision criteria.

*a_ij_* is the lowest ratio from the period of study for alternative *i* with respect to criterion *j*.

*b_ij_* is the average ratio from the period of study for alternative *i* with respect to criterion *j*.

*c_ij_* is the highest ratio from the period of study for alternative *i* with respect to criterion *j*.

Step 5: Define the worst value $f_{j}{}^{-}$ and the best value $f_{j}{}^{*}$ of criterion *j* (financial ratio), where j=1,2,…,n. The worst value $f_{j}{}^{-}$ and the best value $f_{j}{}^{*}$ of criterion j are identified based on the respective criterion j. The financial ratios of CR, EPS, ROA and ROE seek to find the largest value for *a_ij_*, *b_ij_* and *c_ij_*. DAR and DER should be minimized by assigning the smallest value for *a_ij_*, *b_ij_* and *c_ij_*.

Step 6: Compute the evaluation value of criterion *j* for alternative *i* (*S_ij_*) for i=1,…,m, j=1,…,n. $f_{ij}$ refers to the score for alternative *i* with criterion *j*. The normalized fuzzy decision matrix is formed, and the equation to determine the new score of the alternative *i* with criterion *j* is shown below:

(5) $$S_{ij}=\frac{w_{j}(f_{j}*-f_{ij})}{\left(f_{j}*-f_{j}{}^{-}\right)},i=1,\ldots ,m,j=1,\ldots ,n$$

where $w_{j}$ is the weight of criterion *j*.

Step 7: Calculate the utility (*S_i_*), regret (*R_i_*) and VIKOR indices (*Q_i_*) values, i=1,…,m. *v* refers to the strategy of maximum group utility weight, while 1-*v* refers to the individual regret weight. When *v* = 0.5, the strategy could be compromised.

(6) $$S_{i}=\sum_{j=1}^{n}\frac{w_{j}(f_{j}*-f_{ij})}{\left(f_{j}*-f_{j}{}^{-}\right)},i=1,\ldots ,m$$

(7) $$R_{i}=\max\frac{w_{j}(f_{j}*-f_{ij})}{\left(f_{j}*-f_{j}{}^{-}\right)},i=1,\ldots ,m$$

(8) $$Q_{i}=v\frac{\left(S_{i}-S^{*}\right)}{\left(S^{-}-S^{*}\right)}+(1-v)\frac{\left(R_{i}-R^{*}\right)}{\left(R^{-}-R^{*}\right)}$$

where

$S^{*}=\min(S_{i},i=1,\ldots ,m)$

$S^{-}=\max(S_{i},i=1,\ldots ,m)$

$R^{*}=\min(R_{i},i=1,\ldots ,m)$

$R^{-}=\max(R_{i},i=1,\ldots ,m)$

Step 8: Rank the decision alternatives according to the *Q* values [53,60]. The *Q* value is in the range of 0 to 1. A score of zero denotes the most favorable value for a parameter. In contrast, a score of 1 implies the most unfavorable value for a parameter. Select the best alternative by choosing the smallest *Q* value. The alternative with the lowest *Q* value is the closest alternative to the ideal solution.

### 2.3. VIKOR Model

The VIKOR model was introduced to evaluate the financial performance of construction companies [11]. Therefore, the comparison of empirical results was performed between the VIKOR model and the proposed entropy–fuzzy VIKOR model in this study. The VIKOR model is presented as follows [61,62,63]: 

Step 1: Establish the decision matrix for the decision alternatives with respect to the criteria.

(9) $$D=\left[\begin{array}{ccccc} x_{11} & \cdots & x_{1j} & \cdots & x_{1n}\\ \vdots & & \vdots & & \vdots \\ x_{i1} & \cdots & x_{ij} & \cdots & x_{in}\\ \vdots & & \vdots & & \vdots \\ x_{m1} & \cdots & x_{mj} & \cdots & x_{mn} \end{array}\right]$$

where $x_{ij}$ represents the *j*th criterion value of the *i*th alternative of the initial matrix *D*, i=1,…,m, j=1,…,n. *m* refers to the number of decision alternatives. *n* refers to the number of decision criteria.

Step 2: Define the worst value $f_{j}{}^{-}$ and the best value $f_{j}{}^{*}$ of criterion *j* (financial ratio), where j=1,2,…,n. The worst value $f_{j}{}^{-}$ and the best value $f_{j}{}^{*}$ of criterion *j* are identified based on the respective criterion *j*.

Step 3: Compute the evaluation value of criterion *j* for alternative *i* (*S_ij_*) for i=1,…,m, j=1,…,n. $f_{ij}$ refers to the score for alternative *i* with criterion *j*. The normalized decision matrix is formed, and the equation to determine the new score of the alternative *i* with criterion *j* is shown below:

(10) $$S_{ij}=\frac{w_{j}(f_{j}*-f_{ij})}{\left(f_{j}*-f_{j}{}^{-}\right)},i=1,\ldots ,m,j=1,\ldots ,n$$

where $w_{j}$ is the weight of criterion *j*.

Step 4: Calculate the utility (*S_i_*), regret (*R_i_*) and VIKOR indices (*Q_i_*) values, i=1,…,m. *v* refers to the strategy of maximum group utility weight, while 1-*v* refers to the individual regret weight. When *v* = 0.5, the strategy could be compromised.

(11) $$S_{i}=\sum_{j=1}^{n}\frac{w_{j}(f_{j}*-f_{ij})}{\left(f_{j}*-f_{j}{}^{-}\right)},i=1,\ldots ,m$$

(12) $$R_{i}=\max\frac{w_{j}(f_{j}*-f_{ij})}{\left(f_{j}*-f_{j}{}^{-}\right)},i=1,\ldots ,m$$

(13) $$Q_{i}=v\frac{\left(S_{i}-S^{*}\right)}{\left(S^{-}-S^{*}\right)}+(1-v)\frac{\left(R_{i}-R^{*}\right)}{\left(R^{-}-R^{*}\right)}$$

where

$S^{*}=\min(S_{i},i=1,\ldots ,m)$

$S^{-}=\max(S_{i},i=1,\ldots ,m)$

$R^{*}=\min(R_{i},i=1,\ldots ,m)$

$R^{-}=\max(R_{i},i=1,\ldots ,m)$

Step 5: Rank the decision alternatives according to the *Q* values. The *Q* value is in the range of 0 to 1. A score of zero denotes the most favorable value for a parameter. In contrast, a score of 1 implies the most unfavorable value for a parameter. Select the best alternative by choosing the smallest *Q* value.

The VIKOR model has limitations in the setting weight of the decision criteria because the weight is subjectively judged by the decision maker. In this study, equal weight was applied to the financial ratios for the computation of the *Q* value.

## 3. Empirical Results

**Figure 1:**
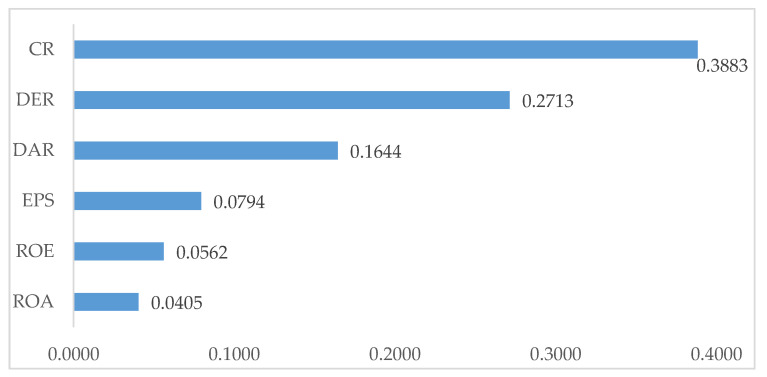
Weights of financial ratios for the performance evaluation of construction companies.

Based on the entropy weighting method in the first stage, the weights of financial ratios for the performance evaluation of construction companies are presented in Figure 1.

As presented in Figure 1, CR (0.3883) obtained the largest weight among the other financial ratios, followed by DER (0.2713), DAR (0.1644), EPS (0.0794), ROE (0.0562) and finally ROA (0.0405). Based on the analysis using the entropy weight method, CR, DER and DAR were the most influential financial ratios to be considered for the performance evaluation of construction companies in this study.

Table 2 presents the fuzzy decision matrix of the construction companies with respect to financial ratios.

Based on the fuzzy decision matrix, the worst $f_{j}{}^{-}$ and the best $f_{j}*$ of alternatives with respect to each criterion were identified. Table 3 presents the worst $f_{j}{}^{-}$ and the best $f_{j}*$ values with respect to each criterion.

In this paper, the financial ratios of ROE, ROA, EPS and CR needed to be maximized. On the other hand, DER and DAR needed to be minimized. Based on Table 3, the best $f_{j}*$ for CR, DAR, DER, EPS, ROA and ROE were 40.845, 393.668 and 1044.293; 0.000, 0.001 and 0.002; 0.000, 0.001 and 0.002; 0.094, 0.248 and 0.498; 5.755, 12.112 and 23.721; and 12.438, 23.787 and 37.165, respectively. In contrast, the worst $f_{j}{}^{-}$ for CR, DAR, DER, EPS, ROA and ROE were 0.116, 1.076 and 1.200; 0.667, 0.745 and 0.811; 2.003, 3.079 and 4.302; 0.000, 0.015 and 0.038; 0.014, 0.297 and 1.322; and 0.015, 0.534 and 2.397, respectively. According to Equation (5), the new score of the alternative *i* with criterion *j* was calculated. Next, the normalized fuzzy decision matrix for the companies with respect to financial ratios is depicted in Table 4.

Based on the normalized fuzzy decision matrix, the triangular fuzzy numbers (TFNs) to measure the construction companies were identified, and the results are summarized in Table 5. The values of utility (*S_i_*) and regret (*R_i_*) were determined by exploiting Equations (6) and (7), respectively.

After that, the values of $S^{*},\;S^{-},\;R^{*}\;\mathrm{and}\;R^{-}$ were determined and tabulated in Table 6.

Based on the result, $S^{*}$= 0.07189, 0.10573 and 0.12119; $S^{-}$= 0.94510, 0.90708 and 0.85570; $R^{*}$= 0.03930, 0.07757 and 0.07856; and $R^{-}$= 0.38831, 0.38831 and 0.38831. In this study, the weight of maximum group utility *v* was assumed to be 0.50 to perform the performance analysis of construction companies. *v* denotes the weight of the strategy “of the majority of criteria”, and it also plays a vital role in the ranking of the alternatives [26].

Table 7 presents the entropy–fuzzy VIKOR scores and optimal ranking for the construction companies. The values of *Q_i_* were determined by using Equation (8). Based on the obtained entropy–fuzzy VIKOR scores (*Q_i_*), the alternative with the smallest *Q_i_* value was specified to be the best construction company.

As shown in Table 7, the values of entropy–fuzzy VIKOR scores (*Q_i_*) and the ranking of the companies were identified based on the proposed model. The *Q_i_* ranged from 0.090 to 0.998 according to the optimal solution of the proposed model that integrates the entropy weight and fuzzy VIKOR model. According to the proposed entropy–fuzzy VIKOR model, the decision alternative with the lowest *Q* value was determined as the best alternative or best construction company. Table 7 shows the results and findings generated by the proposed entropy–fuzzy VIKOR model. The ranking depicts that the best construction company in terms of financial performance was ECONBHD, followed by GADANG, KIMLUN, DKLS, KERJAYA, PTARAS, MITRA, MELATI, PRTASCO, GBGAQRS, BREM, HSL, GAMUDA, IJM, CRESBLD, EKOVEST, GKENT, HOHUP, WCT and lastly MUHIBAH. In this study, ECONBHD achieved the lowest value of *Q*, and thus ECONBHD outperformed the other construction companies in terms of financial performance. The results of this study show that the proposed model is applicable to solve the MCDM problems as illustrated by previous studies, such as the selection of sustainable third-party reverse logistics providers [10] and multi-criteria inventory classification problems [64] using the VIKOR model. The ranking of the companies was important in the financial performance evaluation because it helped the companies to identify their ranking among the competitors in the same field for benchmarking purposes [6].

Finally, the comparison of the scores (*Q_i_*) and optimal ranking of construction companies between the VIKOR model and entropy–fuzzy VIKOR model was performed and presented in Table 8.

As shown in Table 8, both the VIKOR model and entropy–fuzzy VIKOR model obtained different *Q* values or scores. Therefore, the optimal ranking for 90% of the construction companies between the VIKOR model and entropy–fuzzy VIKOR model was significantly different. This implies that the integration of entropy and fuzzy approaches in the VIKOR model has a significant impact on the financial performance evaluation and ranking of construction companies. The integration of the entropy method in the proposed model helps to identify the objective weight of financial ratios, which can avoid the subjectivity of weight selection as compared to the VIKOR model. Furthermore, the integration of the fuzzy approach is able to express the fuzziness, uncertainties, vagueness and imprecision in the evaluation of financial performance.

In summary, the proposed entropy–fuzzy VIKOR model helps to determine the weight of the financial ratios as well as the ranking of construction companies. The findings of this study provide insight into the construction companies for benchmarking based on their current financial status and ranking. For example, the low-ranking companies, such as MUHIBAH and WCT, can improve their financial performance based on the top influential financial ratios, namely CR, DER and DAR. Due to the recent impact of the COVID-19 pandemic, the construction companies should understand their current financial status and ranking in order to sustain, improve and compete with other companies in the same sector. Additionally, this study helped to identify the most influential financial ratios in the financial performance evaluation based on the proposed entropy–fuzzy VIKOR model.

## 4. Conclusions

This paper aims to propose an MDCM model, namely, the entropy–fuzzy VIKOR model, to evaluate and compare the financial performance of construction companies. The proposed model consists of two stages. In the first stage, the entropy weight method is proposed to identify the objective weights of the financial ratios, because it can avoid the subjectivity of weight selection. Based on the analysis using the entropy weight method, CR, DER and DAR are the top three influential financial ratios to be considered for the performance evaluation of construction companies in this study. In the second stage, the fuzzy VIKOR model is proposed to evaluate, compare and rank the construction companies. In this paper, the listed construction companies were evaluated with respect to multiple financial ratios of CR, DAR, DER, EPS, ROA and ROE. This study indicates that ECONBHD achieved the lowest value of *Q* compared to the other construction companies. Therefore, ECONBHD was identified as the best construction company in terms of financial performance, followed by GADANG, KIMLUN, DKLS, KERJAYA, PTARAS, MITRA, MELATI, PRTASCO, GBGAQRS, BREM, HSL, GAMUDA, IJM, CRESBLD, EKOVEST, GKENT, HOHUP, WCT and finally MUHIBAH. As compared to the traditional VIKOR model, the integration of entropy and fuzzy approaches in the VIKOR model has a significant impact on the financial performance evaluation and ranking of construction companies.

This study proposed an integrated entropy–fuzzy VIKOR model to evaluate, compare and rank the financial performance of construction companies based on multiple financial ratios. The proposed entropy–fuzzy VIKOR model is able to determine the weight of financial ratios objectively based on the financial data and, therefore, it helps to replace the subjective weight set by the decision maker using the VIKOR model in previous studies. In addition, the incorporation of the fuzzy method helps to express the fuzziness, uncertainties, vagueness and imprecision in the evaluation of financial performance. Due to the recent impact of the COVID-19 pandemic, the analysis of financial performance using the proposed model will help companies to identify improvement plans by understanding their current financial status and ranking, as well as influential financial ratios.

For future research, the integrated entropy–fuzzy VIKOR model could be considered to evaluate the financial performance of different economic sectors in other countries. Furthermore, the application of the proposed model can be extended to other MCDM problems, such as supplier selection.

## Figures and Tables

**Table 1:** Proposed research framework.

Level	
Objective	Evaluation of the Financial Performance of Construction Companies
	
Decision Criteria	Return on equity (ROE)
(Financial Ratios)	Return on asset (ROA)
	Earnings per share (EPS)
	Debt to equity ratio (DER)
	Debt to assets ratio (DAR)
	Current ratio (CR)
Decision Alternatives	BREM
(Construction	CRESBLD
Companies)	DKLS
	ECONBHD
	EKOVEST
	GADANG
	GAMUDA
	GBGAQRS
	GKENT
	HOHUP
	HSL
	IJM
	KERJAYA
	KIMLUN
	MELATI
	MITRA
	MUHIBAH
	PRTASCO
	PTARAS
	WCT

**Table 2:** Fuzzy decision matrix of the construction companies with respect to financial ratios.

Companies	CR	DAR	DER	EPS	ROA	ROE
BREM	(2.467, 4.694, 7.132)	(0.077, 0.132, 0.240)	(0.083, 0.157, 0.316)	(0.026, 0.063, 0.130)	(1.745, 5.043, 11.186)	(2.296, 5.664, 12.117)
CRESBLD	(0.615, 1.643, 4.115)	(0.235, 0.276, 0.360)	(0.307, 0.387, 0.563)	(0.037, 0.046, 0.050)	(2.358, 2.770, 3.098)	(3.150, 3.830, 4.123)
DKLS	(37.573, 68.835, 114.062)	(0.003, 0.004, 0.004)	(0.003, 0.004, 0.004)	(0.094, 0.147, 0.185)	(3.732, 5.809, 7.722)	(3.745, 5.832, 7.750)
ECONBHD	(10.872, 393.668, 1044.293)	(0.000, 0.001, 0.002)	(0.000, 0.001, 0.002)	(0.005, 0.020, 0.043)	(4.809, 12.112, 17.601)	(4.818, 12.126, 17.617)
EKOVEST	(0.692, 1.123, 1.837)	(0.205, 0.406, 0.555)	(0.258, 0.783, 1.245)	(0.010, 0.065, 0.192)	(0.785, 5.702, 16.555)	(0.988, 11.907, 37.165)
GADANG	(19.045, 175.893, 409.196)	(0.001, 0.006, 0.020)	(0.001, 0.006, 0.020)	(0.020, 0.043, 0.066)	(3.502, 6.671, 11.924)	(3.572, 6.699, 11.953)
GAMUDA	(1.087, 1.629, 2.054)	(0.357, 0.385, 0.403)	(0.555, 0.626, 0.676)	(0.092, 0.248, 0.498)	(2.970, 7.266, 12.883)	(4.619, 11.857, 20.772)
GBGAQRS	(1.007, 15.843, 67.467)	(0.009, 0.124, 0.237)	(0.009, 0.151, 0.310)	(0.007, 0.020, 0.038)	(1.068, 2.775, 5.957)	(1.287, 3.122, 6.881)
GKENT	(1.275, 1.464, 1.871)	(0.420, 0.562, 0.618)	(0.723, 1.342, 1.621)	(0.082, 0.152, 0.226)	(4.780, 10.416, 14.886)	(12.438, 23.787, 34.063)
HOHUP	(1.931, 2.429, 2.794)	(0.360, 0.431, 0.489)	(0.562, 0.775, 0.957)	(0.005, 0.059, 0.179)	(0.330, 4.892, 14.369)	(0.613, 7.907, 22.441)
HSL	(2.234, 2.764, 3.394)	(0.188, 0.241, 0.308)	(0.231, 0.322, 0.445)	(0.064, 0.084, 0.125)	(4.386, 5.769, 9.699)	(5.744, 7.532, 12.169)
IJM	(1.399, 2.273, 3.011)	(0.305, 0.324, 0.341)	(0.438, 0.480, 0.517)	(0.054, 0.082, 0.111)	(2.144, 3.039, 4.015)	(3.084, 4.518, 6.040)
KERJAYA	(8.762, 65.731, 171.582)	(0.005, 0.013, 0.042)	(0.005, 0.014, 0.044)	(0.030, 0.058, 0.101)	(3.640, 7.323, 11.589)	(3.659, 7.414, 11.688)
KIMLUN	(40.845, 78.293, 102.539)	(0.002, 0.003, 0.006)	(0.002, 0.003, 0.006)	(0.045, 0.057, 0.072)	(5.755, 6.958, 8.172)	(5.792, 6.977, 8.184)
MELATI	(0.508, 14.647, 37.898)	(0.011, 0.046, 0.163)	(0.011, 0.053, 0.194)	(0.013, 0.070, 0.247)	(0.781, 4.426, 15.612)	(0.801, 4.588, 15.782)
MITRA	(0.116, 42.494, 128.804)	(0.002, 0.072, 0.143)	(0.002, 0.082, 0.166)	(0.009, 0.033, 0.052)	(1.451, 5.059, 8.293)	(1.615, 5.358, 8.312)
MUHIBAH	(0.951, 1.076, 1.200)	(0.667, 0.745, 0.811)	(2.003, 3.079, 4.302)	(0.033, 0.153, 0.440)	(0.803, 4.072, 12.201)	(3.108, 14.210, 36.642)
PRTASCO	(5.442, 6.604, 8.688)	(0.046, 0.064, 0.078)	(0.048, 0.068, 0.084)	(0.000, 0.048, 0.076)	(0.014, 7.628, 11.783)	(0.015, 8.189, 12.673)
PTARAS	(7.606, 10.674, 11.892)	(0.094, 0.098, 0.103)	(0.103, 0.109, 0.115)	(0.028, 0.181, 0.405)	(2.021, 10.805, 23.721)	(2.230, 12.002, 26.368)
WCT	(2.125, 7.311, 25.602)	(0.274, 0.385, 0.459)	(0.377, 0.645, 0.847)	(0.001, 0.015, 0.067)	(0.025, 0.297, 1.322)	(0.034, 0.534, 2.397)

**Table 3:** The best $f_{j}*$ and the worst $f_{j}{}^{-}$ values for each criterion function.

Financial Ratios	Best ($f_{j}*$)	Worst ($f_{j}{}^{-}$)
CR	(40.845, 393.668, 1044.293)	(0.116, 1.076, 1.200)
DAR	(0.000, 0.001, 0.002)	(0.667, 0.745, 0.811)
DER	(0.000, 0.001, 0.002)	(2.003, 3.079, 4.302)
EPS	(0.094, 0.248, 0.498)	(0.000, 0.015, 0.038)
ROA	(5.755, 12.112, 23.721)	(0.014, 0.297, 1.322)
ROE	(12.438, 23.787, 37.165)	(0.015, 0.534, 2.397)

**Table 4:** The normalized fuzzy decision matrix for the companies with respect to all financial ratios.

Companies	CR	DAR	DER	EPS	ROA	ROE
BREM	(0.366, 0.385, 0.386)	(0.019, 0.029, 0.048)	(0.011, 0.014, 0.020)	(0.058, 0.063, 0.064)	(0.028, 0.024, 0.023)	(0.046, 0.044, 0.040)
CRESBLD	(0.384, 0.388, 0.387)	(0.058, 0.061, 0.073)	(0.042, 0.034, 0.035)	(0.048, 0.069, 0.077)	(0.024, 0.032, 0.037)	(0.042, 0.048, 0.053)
DKLS	(0.031, 0.321, 0.346)	(0.001, 0.001, 0.000)	(0.000, 0.000, 0.000)	(0.000, 0.034, 0.054)	(0.014, 0.022, 0.029)	(0.039, 0.043, 0.048)
ECONBHD	(0.286, 0.000, 0.000)	(0.000, 0.000, 0.000)	(0.000, 0.000, 0.000)	(0.075, 0.078, 0.079)	(0.007, 0.000, 0.011)	(0.034, 0.028, 0.032)
EKOVEST	(0.383, 0.388, 0.388)	(0.051, 0.089, 0.112)	(0.035, 0.069, 0.078)	(0.071, 0.062, 0.053)	(0.035, 0.022, 0.013)	(0.052, 0.029, 0.000)
GADANG	(0.208, 0.215, 0.236)	(0.000, 0.001, 0.004)	(0.000, 0.000, 0.001)	(0.063, 0.070, 0.075)	(0.016, 0.019, 0.021)	(0.040, 0.041, 0.041)
GAMUDA	(0.379, 0.388, 0.388)	(0.088, 0.085, 0.082)	(0.075, 0.055, 0.043)	(0.002, 0.000, 0.000)	(0.020, 0.017, 0.020)	(0.035, 0.029, 0.026)
GBGAQRS	(0.380, 0.374, 0.364)	(0.002, 0.027, 0.048)	(0.001, 0.013, 0.019)	(0.073, 0.078, 0.079)	(0.033, 0.032, 0.032)	(0.050, 0.050, 0.049)
GKENT	(0.377, 0.388, 0.388)	(0.103, 0.124, 0.125)	(0.098, 0.118, 0.102)	(0.011, 0.033, 0.047)	(0.007, 0.006, 0.016)	(0.000, 0.000, 0.005)
HOHUP	(0.371, 0.387, 0.388)	(0.089, 0.095, 0.099)	(0.076, 0.068, 0.060)	(0.075, 0.064, 0.055)	(0.038, 0.025, 0.017)	(0.053, 0.038, 0.024)
HSL	(0.368, 0.387, 0.387)	(0.046, 0.053, 0.062)	(0.031, 0.028, 0.028)	(0.025, 0.056, 0.064)	(0.010, 0.022, 0.025)	(0.030, 0.039, 0.040)
IJM	(0.376, 0.387, 0.388)	(0.075, 0.071, 0.069)	(0.059, 0.042, 0.032)	(0.034, 0.057, 0.067)	(0.025, 0.031, 0.036)	(0.042, 0.047, 0.050)
KERJAYA	(0.306, 0.324, 0.325)	(0.001, 0.003, 0.008)	(0.001, 0.001, 0.003)	(0.054, 0.064, 0.069)	(0.015, 0.016, 0.022)	(0.040, 0.040, 0.041)
KIMLUN	(0.000, 0.312, 0.351)	(0.000, 0.000, 0.001)	(0.000, 0.000, 0.000)	(0.041, 0.065, 0.074)	(0.000, 0.018, 0.028)	(0.030, 0.041, 0.047)
MELATI	(0.385, 0.375, 0.375)	(0.003, 0.010, 0.033)	(0.001, 0.005, 0.012)	(0.069, 0.060, 0.043)	(0.035, 0.026, 0.015)	(0.053, 0.046, 0.035)
MITRA	(0.388, 0.347, 0.341)	(0.000, 0.016, 0.029)	(0.000, 0.007, 0.010)	(0.072, 0.073, 0.077)	(0.030, 0.024, 0.028)	(0.049, 0.045, 0.047)
MUHIBAH	(0.380, 0.388, 0.388)	(0.164, 0.164, 0.164)	(0.271, 0.271, 0.271)	(0.052, 0.032, 0.010)	(0.035, 0.028, 0.021)	(0.042, 0.023, 0.001)
PRTASCO	(0.338, 0.383, 0.386)	(0.011, 0.014, 0.015)	(0.006, 0.006, 0.005)	(0.079, 0.068, 0.073)	(0.040, 0.015, 0.022)	(0.056, 0.038, 0.040)
PTARAS	(0.317, 0.379, 0.384)	(0.023, 0.021, 0.021)	(0.014, 0.009, 0.007)	(0.056, 0.023, 0.016)	(0.026, 0.004, 0.000)	(0.046, 0.028, 0.017)
WCT	(0.369, 0.382, 0.379)	(0.067, 0.085, 0.093)	(0.051, 0.057, 0.053)	(0.079, 0.079, 0.074)	(0.040, 0.040, 0.040)	(0.056, 0.056, 0.056)

**Table 5:** The triangular fuzzy numbers (TFNs) to measure the construction companies.

Companies	*S_i_*	*R_i_*
BREM	(0.528, 0.558, 0.581)	(0.366, 0.385, 0.386)
CRESBLD	(0.597, 0.631, 0.663)	(0.384, 0.388, 0.387)
DKLS	(0.086, 0.421, 0.477)	(0.039, 0.321, 0.346)
ECONBHD	(0.402, 0.106, 0.121)	(0.286, 0.078, 0.079)
EKOVEST	(0.626, 0.659, 0.645)	(0.383, 0.388, 0.388)
GADANG	(0.327, 0.346, 0.378)	(0.208, 0.215, 0.236)
GAMUDA	(0.599, 0.573, 0.558)	(0.379, 0.388, 0.388)
GBGAQRS	(0.540, 0.574, 0.591)	(0.380, 0.374, 0.364)
GKENT	(0.596, 0.668, 0.683)	(0.377, 0.388, 0.388)
HOHUP	(0.703, 0.677, 0.643)	(0.371, 0.387, 0.388)
HSL	(0.511, 0.585, 0.608)	(0.368, 0.387, 0.387)
IJM	(0.612, 0.635, 0.641)	(0.376, 0.387, 0.388)
KERJAYA	(0.416, 0.449, 0.467)	(0.306, 0.324, 0.325)
KIMLUN	(0.072, 0.436, 0.500)	(0.041, 0.312, 0.351)
MELATI	(0.545, 0.523, 0.512)	(0.385, 0.375, 0.375)
MITRA	(0.540, 0.512, 0.531)	(0.388, 0.347, 0.341)
MUHIBAH	(0.945, 0.907, 0.856)	(0.380, 0.388, 0.388)
PRTASCO	(0.531, 0.524, 0.540)	(0.338, 0.383, 0.386)
PTARAS	(0.482, 0.465, 0.446)	(0.317, 0.379, 0.384)
WCT	(0.663, 0.700, 0.696)	(0.369, 0.382, 0.379)

**Table 6:** The fuzzy values for the grading of alternatives.

$S^{*}$	(0.07189, 0.10573, 0.12119)
$S^{-}$	(0.94510, 0.90708, 0.85570)
$R^{*}$	(0.03930, 0.07757, 0.07856)
$R^{-}$	(0.38831, 0.38831, 0.38831)

**Table 7:** The entropy–fuzzy VIKOR scores (*Q_i_*) and optimal ranking of construction companies.

Companies	Entropy-Fuzzy VIKOR Scores (*Q_i_*)	Optimal Ranking
BREM	0.774	11
CRESBLD	0.828	15
DKLS	0.506	4
ECONBHD	0.090	1
EKOVEST	0.841	16
GADANG	0.384	2
GAMUDA	0.791	13
GBGAQRS	0.768	10
GKENT	0.845	17
HOHUP	0.851	18
HSL	0.789	12
IJM	0.826	14
KERJAYA	0.609	5
KIMLUN	0.505	3
MELATI	0.744	8
MITRA	0.704	7
MUHIBAH	0.998	20
PRTASCO	0.747	9
PTARAS	0.697	6
WCT	0.855	19

**Table 8:** The comparison of the scores (*Q_i_*) and optimal ranking of construction companies between the VIKOR model and entropy–fuzzy VIKOR model.

	Entropy-Fuzzy VIKOR Model		VIKOR Model	
Companies	Scores (*Q_i_*)	Optimal Ranking	Scores (*Q_i_*)	Optimal Ranking
BREM	0.774	11	0.770	12
CRESBLD	0.828	15	0.888	18
DKLS	0.506	4	0.194	1
ECONBHD	0.090	1	0.437	5
EKOVEST	0.841	16	0.833	15
GADANG	0.384	2	0.345	4
GAMUDA	0.791	13	0.676	8
GBGAQRS	0.768	10	0.808	14
GKENT	0.845	17	0.693	10
HOHUP	0.851	18	0.870	17
HSL	0.789	12	0.780	13
IJM	0.826	14	0.866	16
KERJAYA	0.609	5	0.248	3
KIMLUN	0.505	3	0.202	2
MELATI	0.744	8	0.684	9
MITRA	0.704	7	0.573	7
MUHIBAH	0.998	20	0.968	19
PRTASCO	0.747	9	0.697	11
PTARAS	0.697	6	0.521	6
WCT	0.855	19	1.000	20
